# Progress in State Medicine

**Published:** 1899-08-05

**Authors:** 


### Progress in State Medicine.

### PUBLIC HEALTH.

**Soil and Disease.**—The Milroy lectures of 1899,[1] delivered by Dr. G. V. Poore, deal with the earth in relation to the preservation and destruction of contagia. The various pathogenic organisms found in soil are described, and their life history followed. From soil are derived those organisms which excite septicæmia, erysipelas, malignant œdema, and tetanus. It is not possible that these can be eradicated, but apart from accidental inoculation they are not dangerous. They are most common in soils rich in organic matter. Anthrax, although very resistant, dies out of most soils in time, except, perhaps, in tropical countries, where it is indigenous. Man is not, however, infected directly from the soil. Malaria, though it disappears before drainage and cultivation, is a doubtful soil disease. Enteric fever, diarrhœa, and plague are filth diseases. In polluted soils their specific organisms may live, but in virgin soil die rapidly. So-called soil diseases are most rife in crowded towns, proving that cultivation plays no part in their spread, but is, indeed, the corner-stone of prevention. In gardens, excreta, which should be removed from the house daily, are best put just below the surface of freshly-dug ground, which is then planted with cabbages. The farm labourer, who, as a rule, tills his garden in this way, is, from Tatham's tables, 33 per cent. healthier than the rest of the community. Natural manure has a far higher fertilising value than artificial. In Holland, where all domestic excreta are added to the soil, the standard of public health is higher than in this country. The dry or conservancy system of excreta removal is consistent with great cleanliness, it is economical, secures the destruction of contagia, and the enrichment of the soil.

Haden[2] finds in the above-mentioned work of Poore arguments in favour of earth burial as opposed to cremation. Imperishable coffins, brick graves, and vaults should be abolished, and the dead placed in as immediate contact with the soil as possible to hasten resolution and to secure the destruction of pathogenic organisms. To obtain this reform cemeteries should be taken out of the hands of the speculator and placed under state or municipal regulation. Whilst a number of crematoria near a large town must prove a nuisance, there is no statistical evidence to show that the death-rate, zymotic death-rate, or rate from any special disease has ever been affected by burial-grounds, even under the present objectionable conditions. Poore's experiments go to show that pathogenic organisms are rendered innocuous when mixed with humus—that is, soil highly impregnated with animal matter; much more so is this the case when virgin soil is employed, as would be the case in the park land used for cemeteries.

These views are diametrically opposed to those of Thorne Thorne,[3] as enunciated in his address on soil and circumstance in their control of pathogenic organisms. In the case of enteric fever he points out that the materies morbi exposed to sunlight and air on an impenetrable surface may quickly die, but a loose, loamy soil organically polluted and moist will maintain the vitality of the organism, and may lead to the contamination of air or water. The laboratory experiments of Sidney Martin[4] demonstrate the possibility of this fact. His experiments show that organically polluted soil which has been sterilised affords a medium in which the typhoid bacillus can retain its vitality, multiply, and become widely diffused. In such soil the bacillus typhosus continued to live for seven to nine months, but in virgin soils had disappeared by the twenty-third day. In this connection Thorne points out how important it is that the yards and courts of dwellings should be paved. Especially is this the case in towns where midden-privies still exist, and where consequently there is greater danger of contamination of soil by excreta. Paving, too, has a marked deterrent effect upon the growth of micro-organisms by decreasing the temperature of the underlying soil and lessening the amount of moisture. The influence of soil and soil temperature upon the incidence of disease is shown again in Ballard's observation that diarrhœa of epidemic type is most prevalent when, with a slight amount of dampness of the surface soil, the four foot earth thermometer registered 56 deg. C. This again points to the necessity of keeping the soil near houses free from organic pollution, and to diminishing the moisture by efficient paving. It should be noted that Poore, in his remarks, referred to soil under natural conditions and clothed with vegetation. Thorne, on the other hand, is dealing with the soil of city courts or yards, or with humus artificially prepared for laboratory experiments. The opinions of various authorities with regard to the relation existing between soil and the distribution of cancer[5] is of much interest. Haviland says the highest death-rates from cancer are associated with seasonably flooded areas in propinquity with fully formed rivers. The soil is generally alluvium with a subsoil of clay. The districts with the lowest mortality are those on carboniferous limestones, the liassic, oolitic, and cretaceous limestones. He instances the lands about the Ouse, Derwent, and Humber, and certain localities in the Thames Valley as possessing high death-rates from cancer. D'Arcy Power finds it impossible to resist the conclusion that the marshy ground from which rivers rise have some causal relation with the number of cases of cancer found in their neighbourhood. He notes that malaria and cancer, however, appear to occur in inverse ratio to each other. Newsholme gives the following list: The highest cancer death-rates were—London, 2,250 per million; Huntingdonshire, 2,157; Cambridgeshire, 2,012; Sussex, 1,999; Warwickshire, 1,976; the lowest, Monmouthshire, 1,574; Dorset and Buckinghamshire, 1,578; Wiltshire, 1,604; and Cornwall, 1,630. He states that no observations hitherto published demonstrate conclusively the existence of cancer houses. The relationship which undoubtedly exists between cancer prevalence and certain conditions of soil, temperature, and moisture would point to the disease being of a parasitic nature.

[1] Brit. Med. Jour., Mar., 1899. [2] Brit. Med. Jour., May 27, 1899. [3] Lancet, Nov. 6, 1897. [4] Local Govt. Board Rep., 1896-7. [5] The Practitioner, April, 1899.

